# Editorial: Special Issue “Galectins: Structure, Function and Therapeutic Inhibitors”

**DOI:** 10.3390/ijms25073674

**Published:** 2024-03-26

**Authors:** Emilia Maria Pedone, Sonia Di Gaetano, Domenica Capasso

**Affiliations:** 1 Institute of Biostructures and Bioimaging, CNR, Via P. Castellino 111, 80131 Naples, Italy; 2 Department of Physics “Ettore Pancini”, University of Naples Federico II, Via Cinthia 4, 80126 Naples, Italy; domenica.capasso@unina.it

Galectins, β-galactoside-binding proteins, play relevant roles in different biological processes; therefore, they are becoming emerging targets for diagnostic and therapeutic approaches. This Special Issue is focused on the characterization of different members belonging to this family, in particular on Gal-1, Gal-3, Gal-4, and Gal-9, analyzing their potential biological activity in different systems such as cancer and viral infection or their identification as novel markers in various pathologies.

Kazuko Hachisu et al. (contribution 1) demonstrate that Gal-4 is an important regulator of glycosylation in cancer cells, and its expression affects the glycan profile of glycosphingolipids (GSLs) in malignant cancer cells with a high potential for peritoneal dissemination. This particular attention is important, as gastric cancer (GC) is one of the most common cancers, the fourth leading cause of cancer-related deaths worldwide, and its incidence rates are highest in eastern Asia [1]. Peritoneal dissemination is the most common form of metastatic or recurrent GC and a major cause of increased mortality. The correlation with Gal-4 is represented by the fact that this protein is expressed in the epithelial cells of the normal gastrointestinal tract. This is the first study to simultaneously investigate the glycan profiles of cell surface proteins and GSLs of cells with different Gal-4 expression and metastatic potential.

In addition to galectins and sialic acid-binding immunoglobulin-type lectins, the receptors of the immune system that bind carbohydrates also include c-type lectin receptors (CLR), together forming the largest receptor family among the pathogen recognition receptors [2]. Two of the best-studied CLRs are dendritic cell-specific intercellular adhesion molecule-3-grabbing non-integrin (DC-SIGN) and langerin, which recognize various glycan motifs [3,4].

Reshmi Mukherjee et al. (contribution 2) report the specific role of the most abundant Human Milk Oligosaccharide component, 20-Fucosyllactose (20-FL), as inhibitor of the binding with C-Type Lectin DC-SIGN and not with the other C-type lectin receptor, langerin. Previously, they had already shown that 20-FL binds to DC-SIGN but not to langerin. In this paper, they report MD simulation data, supporting the insight that 20-FL directly binds to the CRD of DC-SIGN and ligand-receptor competition assays, showing that 20-FL inhibits the binding of the DC-SIGN to its prototypical ligands, fucose and the oligosaccharide Lewis-B, (Leb), in a dose-dependent way. This study implies that 20-FL can outcompete the Leb for binding to the CRD of DC-SIGN. These specific interactions between Leb and DC-SIGN might be important in shaping the microbiota and immune balance in health, considering the importance of Leb in host–microbe interactions [5] on the mucosal surface, the anatomical localization and expression on the myeloid surface, and the anatomical localization and expression of DC-SIGN on the myeloid subpopulations of immune cells.

Considering the huge demand for new markers and therapies to modulate the course of disease progression and develop better treatment options for individuals affected by neurodegenerative disorders, the review by Sapana Chaudhary et al. (contribution 3) discusses the role of Gals in the causation and progression of neurodegenerative disorders. The correlation between Gals and neurodegenerative disorders is suggested by the role of Gals in modulating neuroinflammation, a common factor and one of the main inducers of neuronal damage and degeneration. The role of Gals in microglia and astrocyte modulation, along with their pro- and anti-inflammatory functions, is described. In addition, the potential use of Gals as a novel therapeutic target for neuroinflammation and restoring tissue damage in neurodegenerative diseases is discussed.

As reviewed by Zaborska et al. (contribution 4), Gal-3 is involved in the pathophysiology of heart failure (HF), particularly for its role in cardiac fibrosis, inflammation, and ventricular alteration. HF is a clinical syndrome with high morbidity and mortality, and its frequency is increasing.

A higher level of Gal-3 suggests a higher risk of all-cause and cardiovascular mortality and a higher risk of complications.

Nevertheless, due to the increasing understanding of the molecular characteristics of Gal-3 and its mechanism of action, in addition to the progress of knowledge about the pathophysiology of HF and the therapeutic approach, it is possible to adapt medical procedures according to the characteristics of the patient population and the time when the disease develops.

Moreover, a deep comprehension of the biology of Gal-3, mainly at the level of the genetics and pathology of HF, allows promising research on Gal-3 as a therapeutic target.

Even though recent results indicate potential clinical applications in humans, the clinical utility of therapeutic strategies directed at high Gal-3 concentrations still needs to be studied.

Different expression patterns in bone marrow stromal cells and in hematopoietic cells are revealed in the paper by Fei et al. (contribution 5), for Gal-1 and -3; in particular, the latter is dynamically regulated by extrinsic factors such as chemotherapy. Inhibition of these galectins in the extracellular compartment by plant-derived carbohydrate inhibitors, such as GM-CT-01 and GR-MD-02, in B-cell precursor (BCP-ALL) cells co-cultured with stromal cells attenuated the migration of the BCP-ALL cells to stromal cells and sensitized human BCP-ALL cells to the chemotherapeutic agent vincristine. In addition, it seems that Gal-1 can compensate, to some extent, for the lack of Gal-3 expression in Gal-3 knockout cells. Overall, the results indicate a complex combined contribution of Gal-1 and Gal-3 to BCP-ALL survival, with different roles for endogenous and stromal-produced galectins. These data indicate that an effective strategy for cancer therapy will be to simultaneously and efficiently block both extracellular and intracellular Gal-1 and Gal-3 with the goal of potentiating the activity of drugs during chemotherapy.

An intriguing study was conducted by Pozder et al. (contribution 6) in which Gal-3 was associated with cardiovascular (CV) events. Gal-3 is known to be an endogenous ligand of Willebrand factor (VWF) and red blood cells (RBCs). They evaluated the binding ability of Gal-3 to RBCs and VWF in different blood groups and simultaneously measured the plasma levels of Gal-3 in different blood groups in two cohorts of patients. Gal-3 was shown to have a higher binding capacity for RBCs and VWF in non-O blood groups, compared with blood group O. In addition, patients with non-O blood groups had substantially lower plasma levels of Gal-3. Finally, the independent prognostic value of Gal-3 for all-cause mortality showed a non-significant trend towards higher mortality in non-O blood groups. The authors concluded that the physical interaction between Gal-3 and blood group epitopes can modulate Gal-3, which may influence its circulating plasma level, affecting its biomarker potential and biological activity.

Gal-9 induces HIV reactivation and contributes to non-AIDS events through inflammaging (contribution 7); for this reason, it is important to assess its levels in HIV-infected individuals to determine their association with HIV viremia and other co-morbidities. In this work, plasma Gal-9 levels were evaluated in viremic and aviremic individuals on first-line antiretroviral therapy (ART). They were assessed for correlation with HIV-1 viral load (VL), CD4 count, and ART duration. The results indicated that plasma Gal-9 levels correlated positively with VL and ART duration and negatively with CD4 count. Total Gal-9 levels were influenced by the duration of ART, decreasing its sensitivity to detecting viremia in people living with HIV on long-term ART. However, the total Gal-9-to-CD4 count ratio results have the potential to detect viremic individuals independent of their treatment duration. The results motivate an in-depth study to develop Gal-9 inhibitors that are capable of hindering its contributing effects to HIV viremia and non-AIDS events.

A comprehensive review by Stojanovic et al. (contribution 8) aims to elucidate the multiple functions of Gal-3, starting with its crucial involvement in viral entry by facilitating viral attack and catalyzing internalization. Gal-3 assumes significant roles in the modulation of immune responses, including the activation and recruitment of immune cells, the regulation of immune signaling pathways, and the orchestration of cellular processes such as apoptosis and autophagy. Gal-3’s impact extends to the viral life cycle, including critical steps such as replication, assembly, and release. Notably, Gal-3 also contributes to viral pathogenesis, demonstrating involvement in tissue damage, inflammation, and elements of viral persistence and latency. A detailed examination of specific viral diseases, including SARS-CoV-2, HIV, and influenza A, highlights the intricate role of Gal-3 in modulating immune responses and facilitating virus adhesion and entry. Furthermore, the potential of Gal-3 as a biomarker for disease severity, particularly in COVID-19, is considered. Insight into the mechanisms and roles of Gal-3 in these infections could pave the way for the development of innovative treatment and prevention options for a wide range of viral diseases.

Liesenhoff et al. (contribution 9) focused their work on the role of Gal-1 in the biological homeostasis of cells and the maintenance of the epithelial phenotype of immortalized human retinal pigment epithelium (RPE) cells in vitro. This work highlights that the lack of Gal-1 reduces cell proliferation and viability in immortalized RPE cells and leads to a more mesenchymal phenotype and to an enhanced expression of sm-α-actin and N-cadherin in immortalized RPE cells; in addition, its deficiency enhances adhesion and reduces migration of RPE cells. Finally, in Gal-1-deficient RPE-19 cells, the expression of Gal-8, but not that of Gal-3, is enhanced.

On the other hand, Pirone et al. (contribution 10) addressed the interaction between Gal-1 and Gal-3 with the selenylated analogue of the Gal inhibitor thiodigalactose, characterized by a selenoglycoside bond (SeDG), and with unsymmetrical diglycosyl selenides (unsym(Se). The proteins were produced heterologously and biophysically characterized. Using ITC, NMR spectroscopy, and MD simulation, interaction studies were performed, and the thermodynamic values were compared and combined with the spectroscopic and computational results. The 3D complexes involving SeDG, when it interacts with Gal-1 and Gal-3, were shown. Altogether, the results gathered will help to identify critical points for the design of new, better-performing, and more specific Gal inhibitors.

Finally, the same research group (contribution 11) aimed to study in detail the interaction between Gal-3 and the LPS of *Pseudomonas aeruginosa* using several complementary methodologies, such as circular dichroism, spectrofluorimetry, and dynamic and static light scattering, and the impact of Gal-3 on the redox potential of *Escherichia coli and P. aeruginosa* cell membranes, as well as the binding between the protein and LPS, using ITC and NMR. This in-depth investigation strengthens the hypothesis of an interaction between Gal-3 and LPS, revealing structural details and providing valuable insights into the formation of these intricate molecular complexes. Taken together, these findings could potentially prompt the design of useful therapeutic drugs to develop agonists and/or antagonists for LPS receptors such as galectins as adjunctive therapy for *P. aeruginosa*.

## References

[1] Sung H., Ferlay J., Siegel R.L., Laversanne M., Soerjomataram I., Jemal A., Bray F. (2021). Global Cancer Statistics 2020: GLOBOCAN Estimates of Incidence and Mortality Worldwide for 36 Cancers in 185 Countries. CA Cancer J. Clin..

[2] Peters K., Peters M. (2021). The Role of Lectin Receptors and Their Ligands in Controlling Allergic Inflammation. Front Immunol..

[3] Valverde P., Martínez J.D., Cañada F.J., Ardá A., Jiménez-Barbero J. (2020). Molecular Recognition in C-Type Lectins: The Cases of DC-SIGN, Langerin, MGL, and L-Sectin. ChemBioChem.

[4] Wang Z., Chen J.Q., Liu J.L., Tian L. (2019). Issues on peritoneal metastasis of gastric cancer: An update. World J. Surg. Oncol..

[5] Ewald D.R., Summer S.C.J. (2018). Human Microbiota, Blood Group Antigens, and Disease. Wiley Interdiscip. Rev. Syst. Biol. Med..

